# Survey for Assessment of a Person’s Legal Consciousness: Development and Preliminary Validation

**DOI:** 10.3390/bs10050089

**Published:** 2020-05-12

**Authors:** Victoria Molotova, Alexander Molotov, Dmitry Kashirsky, Natalia Sabelnikova

**Affiliations:** 1 Department of Psychology, Institute of Psychology and Pedagogics, Altai State Pedagogical University, 55 Molodezhnaya Str., 656031 Barnaul, Russia; nsabelni@mail.ru; 2 Department of Constitutional and International Law, Law Institute, Altai State University, 61 Lenin Pr., 656049 Barnaul, Russia; Lawmaking@yandex.ru; 3 Department of Psychology and Educational Technologies, Institute of Higher and Supplementary Professional Education, Federal Research and Clinical Center of Intensive Care Medicine and Rehabilitology, 25 Bld. 2, Petrovka Str., 107031 Moscow, Russia; psymath@mail.ru; 4 Department of Criminal Procedure and Criminalistics, Law Institute, Altai State University, 61 Lenin Pr., 656049 Barnaul, Russia

**Keywords:** legal awareness, degree of significance of rights and freedoms, legal consciousness questionnaire (LCQ), factor structure, construct validity, internal consistency

## Abstract

The results of the development and preliminary assessment of the psychometric properties of the questionnaire of legal consciousness of a person are presented. Theoretical justification is given for the structure of the questionnaire containing six subscales. One scale relates to the individual’s awareness of constitutional rights and freedoms, enshrined in the Universal Declaration of Human Rights and the Constitution of the Russian Federation (personal, economic, political, cultural and social rights). The other five scales relate to the importance of constitutional rights and freedoms for the subject. The content validity of the tool was confirmed by expert assessments of professional lawyers—specialists in constitutional law—and the results obtained with semi-structured interviews (*n* = 30). The construct validity of the tool was established using exploratory factor analysis and tested by confirmatory factor analysis for student sample (*n* = 100). Cronbach’s alpha indicated high degree of internal consistency of the subscales. Currently, we continue improving the psychometric characteristics of the measure. The questionnaire obtained as a result of this work can be used to assess the level of a person’s legal consciousness development, and in particular, during the professional personnel selection for the state and civil service. The results of large-scale studies carried out with the help of this tool can be implemented in the activities of public authorities in order to improve the legislative process, taken into account by public organizations and authorities involved in the spread of legal education and legal culture in the framework of state policy on the development of legal consciousness and legal culture.

## 1. Introduction

Legal awareness, as a theoretical construct and sociocultural phenomenon, has been the issue of a significant research interest of representatives of various scientific disciplines—philosophy, sociology, ethics, jurisprudence, etc. Psychology does not stand aside, addressing law representation in the individual consciousness. The specifics of the legal awareness, its formation, development, and relevance of research instruments are on the list of issues that modern psychology is trying to research. The law, being one of the regulators of social relations, cannot be separated from the subjects of public life; therefore, the issues of legal awareness cannot be thoroughly investigated and understood without psychological research.

According to the functional-structural concept of A.R. Ratinova [1], legal relationships are reflected in a person’s legal consciousness in a specific way. Legal consciousness has its particular language, a system of concepts and categories, certain criteria and methods of assessment. In other words, the legal consciousness of the individual has a certain cognitive apparatus, its own system of values, methods of practical activity management. Accordingly, we can talk about the three functions of legal consciousness: cognitive, evaluative and regulatory. In the view of A.R. Ratinova, all forms of mental reflection of the legal life of society can be attributed to the legal consciousness [2]. According to M.I. Enikeev [3], legal consciousness can express itself in the motives of legally significant behavior by the manifestation of solidarity with law and general legal principles, guidelines, values, or by the manifestation of legal negativism, or by the denial of legal values developed at a certain stage in the development of society. P.P. Baranov [4] identifies legal feelings, attitudes, legal experience, and legal illusions as basic elements of legal consciousness. O.V. Pristanskaya and E.M. Yutskova [5] indicate the following elements in the structure of legal consciousness: knowledge of law, ideas about law, attitudes and requirements for law, attitude towards the fulfillment of legal prescriptions. In his article V.A. Shchegortsev [6] introduces the four-component model of legal consciousness which includes substantive component (knowledge and ideas of people about legal phenomena), evaluative component (assessment of legal phenomena by people), behavioral component (behavior and activity of people in right-handed situations) and energy (emotions and feelings of people experienced in a meaningful situation). According to O.A. Gulevich [7], sense of justice can be defined as a set of social attitudes to crimes and criminals, law, punishment, law enforcement, judicial and penal systems. Thus, the structure of legal consciousness consists of three components: cognitive (legal concepts, knowledge, cognitive assessment of legal phenomena), affective (emotional assessment of legal phenomena) and behavioral (intention to behave in a certain way in legal situations).

Legal consciousness of the individual is developed as a result of legal socialization. The legal socialization is the internalization of legal norms, legal behavioral patterns acquirement, social responsibility and law-obedience development, and, consequently, the inclusion of these attributes into the system of one’s personal norms and values. Family, educational environment, and other social institutions exert an impact on the legal socialization of the individual and contribute to the legal consciousness development. Thus, legal norms and values are complied with primarily due to the commitment to law, rather than the fear of punishment. Therefore, one of the most important aspects of the modern national and education policy in Russia is the development of the legal norms and values, adopted at the international level and enshrined in the international regulatory acts; development of the law compliance patterns within the younger generation [3]. We consider the legal awareness of the individual as a reflection of legal norms and values in the consciousness of the individual, which involves such phenomena indicating the level of development of the legal consciousness and its basic psychological structure as: awareness of the subject of basic rights and freedoms (objective legal norms), significance of the particular rights and freedoms for the individual, peculiarities of the subject’s behavior and activities within the legal field (compliance, violation or denial of legal norms).

The measurement of the legal consciousness of the individual is of particular relevance. To date, however there is no valid and reliable psychological assessment tool kit to give a holistic quantitative assessment of the level of legal consciousness development. The difficulty in creating such instruments is, first of all, due to the insufficiently developed theoretical and methodological grounds for related applied research. At present, all available instruments are aimed at measurement of certain aspects of legal consciousness: its cognitive component [8,9,10], as well as the emotional and behavioral ones [11,12,13]. However, there are no valid and reliable tools to assess the level of subjects’ awareness of basic rights and freedoms and to identify the degree of significance of the initial legal values which constitute the essential characteristic of the legal consciousness of the person, acting as guidelines for particular behavior and activities in a legally significant situation.

Psychological analysis of the legal consciousness of the individual should be based on the following provisions: (1) the legal consciousness of the individual is more associated with the essence of law, rather than with individual legal norms and laws; (2) while analyzing the structure of a person’s legal consciousness and assessing its qualitative characteristics, the researcher should primarily consider its subjective (appraisal) side, which is expressed in the subject’s personal attitude to the basic legal principles; (3) various spheres of legal consciousness of an individual are directly connected with an objective legal system—branches of law and specific types of legal relations—which are reflected in the international acts and national constitutions. In essence, these legal acts are nothing more than the universal values of the human being officially established by the legislator.

Thus, the goal of the present study is to create a theoretical model and to perform a preliminary assessment of the psychometric properties of the questionnaire designed to study the holistic structure of the legal consciousness of the individual. The developed tool is aimed at measurement of (1) the level of the subject’s awareness of Constitutional human rights and freedoms; (2) the importance for the subject of basic rights and freedoms that underlie the formation of the law and legal regulation of the system, which exist in society at the present level of development of society.

Consequently, the questionnaire is designed to study the holistic structure of the legal consciousness, its cognitive (the awareness of rights, enshrined in regulatory acts), as well as the evaluative and behavioral (the significance of rights) properties. The integral research of the legal consciousness of the individual is conditioned by the theoretical justification of the questionnaire’s structure, with the Constitutional Rights being its measurements. The developed questionnaire is projected to assess the individual’s degree of acquirement of the legal norms, adopted at the international level, which are particularly important for the national legal framework. The new diagnostic tool is capable of defining the legal consciousness qualitative feature and, subsequently, allowing conclusions about the socialization of Russian youth on legal issues.

Research questions of our study were: (1) What are the components of a basic psychological structure of legal consciousness? (2) Do the empirical data gathered with the tool developed on the basis of the model of the psychological structure of legal consciousness fit the theoretical construct of human values, reflected in the Universal Declaration of Human Rights and the Constitution of the Russian?

## 2. Methods

The development of the Legal Consciousness Questionnaire (LCQ) included the following steps: (1) performing theoretical analysis of relevant scientific publications on legal issues and examination of previous research on the development of diagnostic tools for measuring individual’s legal consciousness; (2) analyzing the Universal Declaration of Human Rights and the Constitution of the Russian Federation; (3) designing and conducting of semi-structured interviews; (4) developing the structure of the new questionnaire and making an expert assessment of items; (5) performing empirical validation of the test-analysis of the structure and internal consistency of subscales of the questionnaire as well as qualitative analysis of obtained data.

The questionnaire is based on legal axioms connected with values and norms that are objectively enshrined in the Universal Declaration of Human Rights [14] by the world community and subsequently reflected in constitutional rights and freedoms of citizens of the Russian Federation [15]. Since these legal acts express the rights and freedoms of citizens existing at a certain stage of the cultural and historical development of society, as a kind of “ideal form” (L.S. Vygotsky) for the formation of a person’s legal awareness, they serve as an important objective criterion for studying the structure of a person’s legal consciousness which is the result of the internalization of the rights and freedoms accepted in society [16]. Thus, the first step of the present research was devoted to the theoretical analysis of the Universal Declaration of Human Rights and the Constitution of the Russian Federation aimed at reconstructing the basic spheres of legal regulation governing the legal relations of people in modern Russian society.

Next, we designed and conducted a semi-structured interview (*n* = 30) aimed at identification of the qualitative characteristics of the legal consciousness manifested in legal literacy (awareness), the level of law understanding, as well as varying degrees of the significance of constitutional rights and freedoms for the individual. In that way the law was assumed as empirical phenomenon existing at the level of consciousness and behavior of the individual. 

Graduate students of non-legal specialties of Altai State University took part in the interviews. The total number of tested was 30 people (12 male adolescents, 18 female adolescents), 17 of them were the students of the faculty of psychology and pedagogy, 4 persons were the students of the faculty of journalism, 6 persons were the students of the faculty of sociology, 3 persons were the students of the faculty of geography. The participants’ ages ranged from 21 to 23 years old.

The study was conducted on the basis of the faculty of psychology of Altai State University. Students were announced about the upcoming study through social networks. Interviewing was voluntary and was carried out in a specially designated comfortable room of the psychological laboratory of Altai State University that meets the requirements and standards for the premises preparation and conduct of psychological research. The organizers received a written consent of each respondent to take part in the study, in compliance with the requirements of current Russian Federation legislation and international ethical standards in psychological research.

The interview design, the pilot study, the research data collection and analysis covered several stages from October 2017 to May 2018. The interview with one respondent lasted 2.5 h on average, and the analysis of the data results took up about 16 h. 

Conducting the interview aimed at studying a person’s legal consciousness requires in-depth knowledge of the research subject from the interviewer, i.e., about the law. The interview was conducted by V.V. Molotova, one of the authors of the article, who obtained not only a degree in psychology, but also in law. Moreover, the interviewer has got an extensive experience as a practicing advocate.

Table 1 gives descriptions of the questions reference, the examples of the interview questions and the examples of the processed data results.

The choice of the interview was grounded by the fact that we aimed at identifying new, previously neither selected nor studied properties of personality consciousness by psychologists. In our opinion, it is precisely this type of research design makes it possible to identify many semantic nuances of the phenomenon under study by comparing and summarizing the views of interlocutors on questions posed by the interviewer [17].

An interview is a very flexible tool that allows you to get a description of the experience of research participants in their own words in the process of communication. Moreover, although the set of questions asked was strictly determined by us, it nonetheless varied depending on the responses received. Therefore, the interview we used was “semi-structured”, i.e., customizable taking into account the individual characteristics of the interviewee, but without compromising the quality of the information collected.

The first version of the Legal Consciousness Questionnaire (LCQ) was developed on the basis of further generalizations of the interview responses. Thereafter the lawyers—experts in international and constitutional law—evaluated the draft in order to confirm the adequacy of item wording to the researched concept.

Finally, empirical validation of LCQ was carried out by means of the exploratory factor analysis and tested by confirmatory factor analysis using a student sample (*n* = 100). The internal consistency of the questionnaire scales was confirmed using Cronbach’s Alpha coefficient.

## 3. Results

### 3.1. Theoretical Basis for the Scale Structure

The analysis of the Universal Declaration of Human Rights [14] and the current Constitution of the Russian Federation [15], as well as the works of leading experts in the field of the theory of international and constitutional law [18,19] allowed us to identify five basic areas of legal regulation: (1) Personal Rights (the right to life and health, freedom of speech and thought, freedom of religion, freedom of conscience, the right to privacy, the right to freedom of movement); (2) Economic Rights (the right to own, use and dispose of property on the basis of the right of ownership, the right to freely dispose of their ability to work, the right to run business activities); (3) Political Rights (the right to elect and be elected to the authorities, the right to participate in rallies and processions, the right to join trade unions, freedom of peaceful assembly and association, the right of access to public service); (4) Social Rights (the right to social security, namely free medical care and education, pensions, guarantees and benefits for the poor, disabled, orphans, minors); (5) Cultural Rights (the right to freedom of creativity, freedom to getting education, access to cultural heritage). Thus, the results of the analysis of the legal regulation sphere constituted a five-factor hypothetical model of the questionnaire based on theoretical and legal ideas about the principles and mechanisms of legal regulation in the basic spheres of life and social interaction of the individual. The obtained structure of the questionnaire gives the researcher the possibility of a holistic assessment of the legal consciousness of the individual.

In addition to this, a scale of awareness of international rights and freedoms related to the cognitive component of personal sense of justice was added to measure the level of the legal literacy of the subjects. Introducing this scale, we made it possible to compare the subjects’ knowledge of certain rights and freedoms with their significance for the subject. These comparisons are extremely important for understanding the of the individual’s legal consciousness.

The research question of our study was: Do the empirical data gathered with the tool developed on the basis of the model of the psychological structure of legal consciousness fit the theoretical construct of human values, reflected in the Universal Declaration of Human Rights and the Constitution of the Russian Federation?

### 3.2. Development of the Item Pool and Extension of the Scale Structure

The exact items for measuring a priori five factor definition were also developed on the basis of analysis of the Universal Declaration of Human Rights, the current Constitution of the Russian Federation and publications of the leading experts in constitutional law. These items were included in a semi-structured interview (*n* = 30) aimed at assessment of the legal literacy (awareness), the level of law understanding, and degree of the significance of constitutional rights and freedoms for the individual. The respondents were asked to express their attitude to the existing constitutional norms, to indicate how necessary and meaningful they are in social interaction and whether the subjects considered acceptable and possible for them to cancel or limit the categories of rights and freedoms established by law.

The analysis of the data obtained by the semi-structured interview allowed us to formulate the questionnaire items in a way clear to an average person. Meanwhile, the modified wording could distort the essence of statutory rights and freedoms of the citizens. Therefore, the experts in the field of constitutional law were invited to assess the adequacy of the test questions. As a result of this work, a questionnaire containing 56 items was developed.

Table 2 shows an example of an algorithm for generalizing and categorizing interview data of the fourth topic block (i.e., the importance of rights and freedoms), that were subsequently transformed into questionnaire content.

### 3.3. The Measure

The developed Legal Consciousness Questionnaire (LCQ) contains 56 items representing six subscales: (1) Awareness of Rights and Freedoms; (2) Personal Significance of Personal Rights and Freedoms; (3) Personal Significance of Economic Rights and Freedoms; (4) Personal Significance of Political Rights and Freedoms; (5) Personal Significance of Social Rights and Freedoms; (6) Personal Significance of Cultural Rights and Freedoms. According to the instructions, the subject was asked to familiarize oneself with these statements and express his agreement with them on a 7-item Likert scale. We provide the examples of items relating to the questionnaire subscales questionnaire below.

Subscale 1. Awareness of Rights and Freedoms (10 items) assesses the respondent’s awareness of one’s rights and freedoms (whether a person hears about them for the first time or, on the contrary, has come across these formulations before and has a fairly complete comprehension of them). The statements relating to this scale are divided into two types (2 statements for each of the five areas of legal regulation). The first type of statements is formulated very generally and does not disclose the content of legal regulation sphere proposed for evaluation, giving a researcher an opportunity to reveal the subjects’ general level of understanding the relevant categories. For example, “I am well aware of my personal rights” (item 1, the sphere of regulation of personal rights), “I am well aware of my economic rights” (item 17, the sphere of regulation of economic rights), etc. The second type of statements relates directly to the text of the Universal Declaration of Human Rights and the Constitution of the Russian Federation and specifies the content of each of the five areas of legal regulation. For example, “I know well that everyone has the right to life and health, freedom of speech and thought, freedom of religion, freedom of conscience, the right to privacy, the right to freedom of movement” (item 2, the sphere of personal rights regulation), “I am well aware that every person has the right to own, use and dispose of property on the basis of the property rights, the right to freely dispose of his ability to work, the right to conduct entrepreneurial activities” (items 18, the sphere of regulation of economic rights), etc.

Subscales 2–5 evaluate subject’s attitude to the content of specific rights related to various areas of legal regulation and social interaction. Each of these scales contains five types of statements which relate to:

(a) significance for the subject of a particular group of rights and freedoms (for example, “it is important for me that no one can interfere in my private and family life, violate the privacy of correspondence, telephone conversations);

(b) willingness to restrict the right in order to serve the public good (for example, to restrict the personal right to freedom of thought and speech, and give a permission for censorship on the Internet in order to save children from harmful information);

(c) willingness to restrict one’s rights in return for the material remuneration or social guarantees (for example, waive the right to elect representatives to government bodies for material remuneration, or abandon the ownership of real estate in favor of a contract of social renting real estate, decent wages, free medical care and job security);

(d) willingness to waive rights or restrict rights in favor of a more significant category of rights (for example, waive the right to free medical care or the right to receive a pension in order to guarantee the smooth running of business activities);

(e) the inadmissibility of the restriction of rights and freedoms, or the denial of certain rights and freedoms (the subject under no circumstances considers the option of restricting one’s own right or waiving it).

Subscale 2. Personal Significance of Personal Rights and Freedoms (14 items) assesses the subject’s attitude to the content of personal rights and freedoms. For example, “It is important for me that people are born with equal rights and freedoms, regardless of family and conditions in which they were born” (item 3), I am ready not to go abroad and stay in Russia if the state provides me with everything I need and creates good conditions for my life (item 7), etc.

Subscale 3. Personal Significance of Economic Rights and Freedoms (4 items) evaluates the subject’s attitude to the content of economic rights and freedoms. For example, “I am ready to give up the ownership of real estate if the state creates a decent standard of living for me and gives me social guarantees” (free education and medicine, decent housing, high-paid work, decent pension, etc.) (item 20), “Under no circumstances I am willing to give up ownership or limit my ownership” (item 21), etc.

Subscale 4. Personal Significance of Political Rights and Freedoms; (8 items) evaluates the subject’s attitude to the content of political rights and freedoms. For example, “It’s important for me that I can elect representatives to state bodies and to be elected to power” (item 28), I am ready to give up the right to elect the authorities for remuneration (item 29), etc.

Subscale 5. Personal Significance of Social Rights and Freedoms; (6 items) assesses the subject’s attitude to the content of social rights and freedoms. For example, ”I am ready to give up the right to freedom of creativity, and I am ready to write poems, songs, books, paintings within the framework of ideologies established by the state, if it affects positively my material well-being and provides me with significant social guarantees” (item 40), ”Under no circumstances I am ready to give up my cultural rights” (item 43), etc.

Subscale 6. Personal Significance of Cultural Rights and Freedoms. (11 items) evaluates the subject’s attitude to the content of cultural rights and freedoms. For example, ”It’s very important for me that I have the right to free education and health insurance” (item 46), “I am ready to give up the right to receive a pension if I can earn so much that in the future I will have enough for a decent ageing”(item 50), etc.

The developed measure was further subjected to factorization, and the evaluation of the internal consistency.

### 3.4. Sample and Procedure

One hundred undergraduate students (60 males and 40 females) participated in the study. Ages ranged from 17 to 20 (*M = 18.2*, *SD = 0.57*). Participants were invited to take part in the study, being confirmed that they were free to withdraw at any time. Participants were debriefed on the nature of the research upon completion and returning the paper-and-pencil survey.

### 3.5. Construct Validity and Internal Consistency of the Subscales

The Kaiser-Meier-Olkin Measure of Sampling Adequacy and the Bartlett sphericity test indicated the suitability of the data for the structure detection (KMO = 0.705, χ2 = 5639.672, df = 1540, *p* ≤ 0.0001).

An exploratory factor analysis (EFA) was carried out (principal component method, Varimax axis rotation) for empirical confirmation of the questionnaire structure. It generally confirmed the six-factors that had a saturation of 61.76% of the total variance (Table 3).

The results of EFA are shown in the Table 4.

However, some items of the initial version of the questionnaire had insufficient loads on extracted factors. These items were dropped and the measure was reduced to 34 statements which formed six subscales: Subscale 1. Awareness of Rights and Freedoms Subscale contained 10 items (1, 2, 17, 18, 26, 27, 36, 37, 44, 45); Subscale 2. Personal Significance of Personal Rights and Freedoms Subscale—5 items (4R, 6R, 7R, 10R, 11); Subscale 3. Personal Significance of Economic Rights and Freedoms Subscale—5 items (20R, 21, 23R, 24R, 25); Subscale 4. Personal Significance of Political Rights and Freedoms Subscale—5 items (4R, 6R, 7R, 10R, 11); Subscale 5. Personal Significance of Social Rights and Freedoms was composed of 4 items (28, 29, 32, 39); and Subscale 6. Personal Significance of Cultural Rights and Freedoms consisted of 5 items (47R, 48R, 53, 54, 55). Numbers of items are presented as they were given in the initial version of the tool (Supplementary Materials). The final version of the questionnaire is shown in the Supplementary Materials.

**Figure 1:**
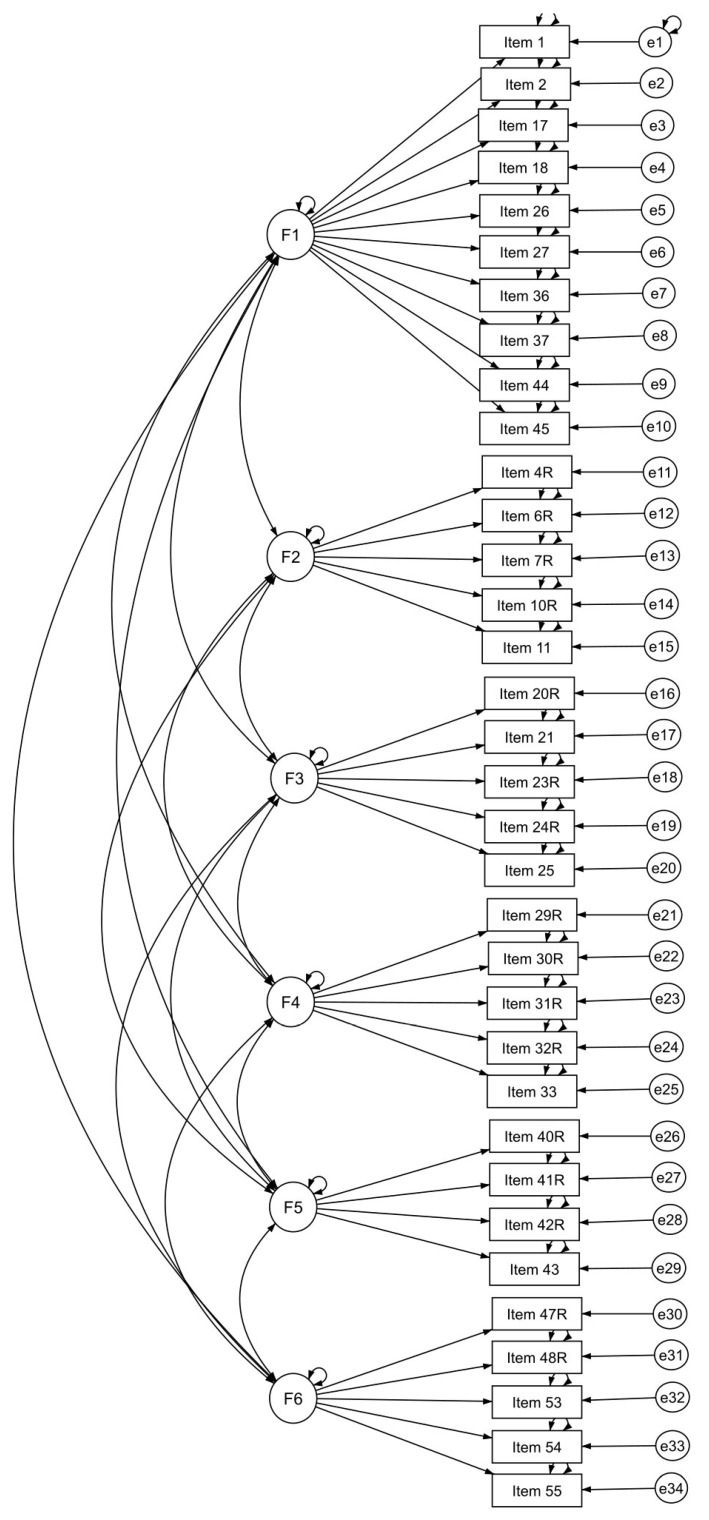
A priori model of the questionnaire structure with 6 factors.

Next, we evaluated the questionnaire factor structure (34 items) by means of Confirmatory Factor Analysis (CFA) (*n* = 100). A priori model is shown in Figure 1.

**Figure 2:**
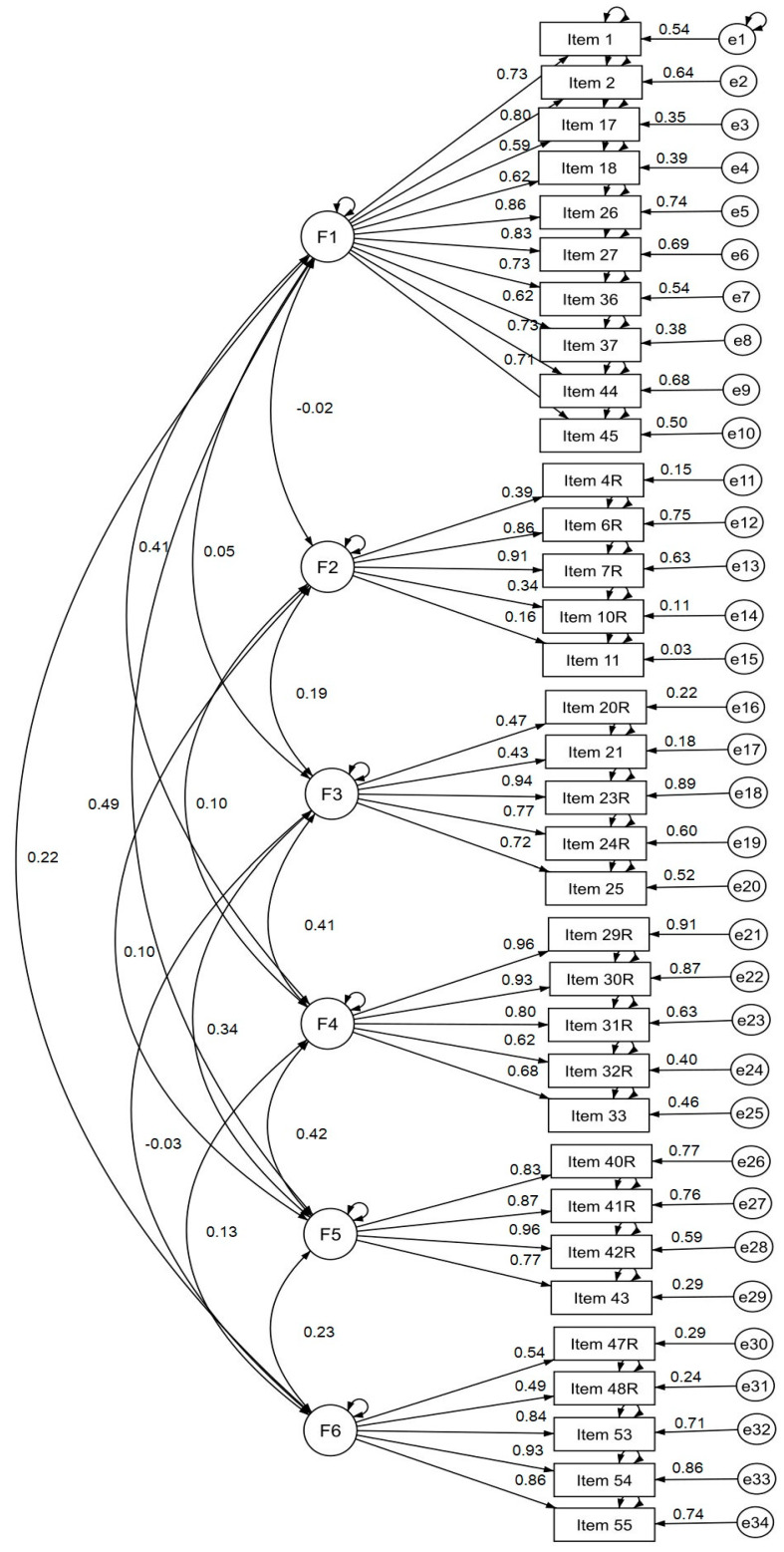
A posteriori six-factor model of the survey.

Next, factor structure of newly developed questionnaire was assessed using CFA (*n* = 100). Statistical indicators showed an acceptable model fit: χ2 = 748.89, df = 455, *p* ≤ 0.0001; χ2/df = 1.646 < 2, CFI = 0.90, RMSEA = 0.08, LO 90 = 0.07, HI 90 = 0.09, TLI = 0.87. A posteriori six-factor model of the Survey is presented in Figure 2.

Thus, the factor solution proved the appropriateness of the developed tool for assessing the significance of rights and freedoms for the subject, the awareness of the rights and freedoms regulated by the laws established in society.

Subsequently, Cronbach’s’ Alpha coefficient was calculated to assess the internal consistency of the LCQ subscales. The obtained results indicate a high consistency of questionnaire scales: (1) Awareness of Rights and Freedoms; (α = 0.92), (2) Personal Significance of Personal Rights and Freedoms (α = 0.75), (3) Personal Significance of Economic Rights and Freedoms (α = 0.84), (4) Personal Significance of Political Rights and Freedoms (α = 0.91), (5) Personal Significance of Cultural Rights and Freedoms (0.93), (6) Significance of Social Rights and Freedoms (α = 0.88).

The obtained coefficients indicated high LCQ subscales consistency.

## 4. Discussion and Conclusions

Human rights and freedoms are a significant achievement of legal thought and a guideline in the development of a modern legal culture not only in Russia but throughout the civilized world. Democratic and legal culture is the priority aspect of the basic culture of the individual and guidelines for upbringing. The development of legal literacy and legal consciousness of citizens is one of the state policy directions of the developed countries.

Legal consciousness research is interdisciplinary in nature that emphasizes its special relevance. A comprehensive study of legal consciousness is impossible without taking into account its psychological aspects. Law is capable of turning from abstraction into a powerful foundation of social life only when it is appropriated by the person, when it becomes a value guide and a measure of an individual’s behavior, the bearer of individual legal consciousness.

The Universal Declaration of Human Rights, as well as the constitutions of states is an objective expression of law at a certain cultural and historical stage of societal development, a “zone of proximal development” of legal consciousness, and a guideline for the development of legal culture for future generations.

Understanding and awareness of the basic legal principles and provisions enshrined at the international level by a person, as well as ones subjective attitude to them, is an important objective criterion that allows you to assess the specifics of the legal consciousness of a particular person.

The aim of the study was to develop an instrument for assessment of the individual’s legal awareness including the awareness of the rights and freedoms adopted by the society, as well as the significance of basic rights and freedoms for the subject that form the basis of legal regulation accepted in the society. Therefore, we performed a preliminarily analysis of the Universal Declaration of Human Rights and the current Constitution of the Russian Federation in order to highlight the basic rights and freedoms of the citizens, which represent an objective (“intrapsychic” according to L.S. Vygotsky) form of legal consciousness. As a result, five basic constitutional rights and freedoms were identified and considered to be five hypothetical factors of the model. Those 5 factors together with the factor named Awareness of Constitutional Rights and freedoms were accounted for a six-factor questionnaire model.

Thereafter, the items of the preliminary questionnaire version were developed and subjected for discussion by means of a semi-structured interview. The interview clearly showed that ordinary people had difficulties in understanding professional lawyers’ wording of the items, which were taken directly from the Constitution. Therefore, we made a decision to correct items’ wording and make them comprehensible for an average person with losing no “letter and spirit of the law” contained in the Universal Declaration of Human Rights and the current Constitution of the Russian Federation. Next, the items were assessed by two professional lawyers—experts in constitutional law. As a result of this work, Legal Consciousness Questionnaire (LCQ), consisting of 56 items, was developed.

Subsequently, the questionnaire was empirically validated. The proposed factor structure was generally confirmed by means of EFA and CFA. However, some items of the first LCQ version were dropped due to low loadings or double loadings. CFA confirmed the construct validity of the questionnaire in general, demonstrating quite satisfactory results of matching the model with empirical data. Nevertheless, we will continue improving the model compliance indexes, first of all, by increasing the sample size, which should lead to a change in the χ2 statistics and the RMSEA index in the direction of their more optimal indicators.

Summing up, the present research introduced a theoretical model of the Legal Consciousness Questionnaire (LCQ) and performed its preliminary empirical validation. The conducted study should be considered as the first step in the development and validation of the valid and reliable measure of person’s legal consciousness.

Currently, the work on improving the psychometric characteristics of the LCQ measure is in progress. One of the possible research directions is expanding the list of items and re-wording the dropped items by splitting them into smaller ones. In our opinion, some of the dropped items were incomprehensible to the subjects or understood ambiguously. Having provided the objective discrepancies between law as a theoretical construct and as an empirical phenomenon, we will try to reduce them in regard to the measure under research.

Further research is also planned to validate the questionnaire on wider variety of samples. In particular, we have started to study the legal awareness of students enrolled in Law Institute of Altai State University and the other university schools. Further study will also include different community samples.

The questionnaire obtained as a result of this work can be used to assess the level of a person’s legal consciousness development, and in particular, during the professional personnel selection for the state and civil service. The results of large-scale studies carried out with the help of this tool can be implemented in the activities of public authorities in order to improve the legislative process, taken into account by public organizations and authorities involved in the spread of legal education and legal culture within the framework of state policy on the development of legal consciousness and legal culture.

## Figures and Tables

**Table 1:** Interview structure *.

No.	Reference of Questions	Examples of the Interview Questions	Generalization and Categorization Results
1.	Introductory questions that allow you to establish contact with the subject, form an atmosphere of trust, get general information about the person, area of interest, social activity, citizenship position	“Do you like the city you live in?”, “Is an individual able to influence the development of comfort in one’s city/country in your opinion?”, “Who is more responsible for the quality-of-life in society, citizens or authorities?”, “Do you take part in public life/political life?”, “Have you had to study law?”, “Have you ever had to defend your violated rights?”	There has been determined a general concept of the respondent’s personality, his or her willingness to participate in an interview, assessment of emotional involvement in the conversation, interest in the topic of conversation, interest in social and political institutions, his or her own role and position in social interaction, as well as one’s general level of cultural development.
2.	Questions aimed at clarifying the semantic content for the subject of the concepts of “law” and “legal act”, understanding the functions of law, the proportionality of law with other regulators of social relations (morality, religion, etc.)	“How do you think the concept of law and the concept of legal act are related?”, “How does the legal act differ from law?”, “What are the main functions of law/legal act?”, “How do legal norms differ from moral/ethical/religious ones?“ “Can the legal and moral norms coincide in their content?”, “Does the law affect the formation of moral norms?”, “Do moral norms influence the formation of legal ones?”, “Does the law affect the development of society?”, “Does society affect the development of law?”, “Does law alter with a change in social relations?”	There has been made an assessment of the general level of a respondent’s cultural development in the field of legal relations, the degree of knowledge of the proposed concepts, the ability to reason, generalize, compare. The level of legal comprehension has been assessed as well.
3.	Questions reflecting the respondent’s awareness of the basic principles of the state structure, its legal system, the legal status of an individual, fundamental rights and guarantees enshrined in the International Acts (personal, political, social, economic, cultural rights and freedoms, etc.)	“Have you ever heard of the principle of separation of powers?”, “How do you understand what a representative government body is?”, “What are the known to you forms of government/political structure/territorial structure/economic regime?”, “What human rights and freedoms are known to you?”, “Please name the main categories of rights,” “How do you understand the content of these categories of rights?”	There has been made an assessment of the level of theoretical generalizations that the respondent demonstrates during the interview, a literal or abstract concept of the categories proposed for comprehension, an assessment of the respondent’s general awareness of the concepts being discussed, as well as the semantic content of the concepts and categories under consideration.
4.	The questions directed on determining the significance for the interviewee of the basic guaranteed rights and freedoms (personal, political, economic, social, cultural), satisfaction with these rights, willingness to refuse or proportionately restrict certain rights and freedoms, determining the level of more significant and less significant rights and freedoms.	“What are the most important rights and guarantees out of the list we have just discussed?”“What are the most significant and least significant rights for you, justify your position”. In addition, respondents were given a list of existing human rights with a request to evaluate and comment on them, to express a judgment on how necessary and significant this norm is in society and for him or her personally; specify what values are protected by this legal norm, whether this category of rights and freedoms can be deprived, or limited.	There has been revealed the attitude of respondents to different categories of human rights, depending on those social values that are “protected” by a particular category of rights, namely personal, political, economic, social, cultural ones. The results of processing and analysis of this part of the interview made it possible to single out a criterion for the significance of rights and freedoms for the respondent—the willingness to refuse guaranteed rights and freedoms, the willingness to proportionally limit guaranteed rights and freedoms in favor of more significant ones.
5.	Questions reflecting overall emotional assessment of law as a sociocultural phenomenon, a personal assessment of the effectiveness of existing norms, an assessment from the standpoint of justice.	“Do you think that the provisions on human rights are mainly respected?”, “Do you think the laws are fair?” “Give an example of social relations in which human rights are not respected”, “Give an example of an unfair law”, “Is law needed in a highly developed society, in your opinion?”	There have been revealed the differences in the emotional assessment of law as a sociocultural phenomenon, i.e., negative, neutral, or positive attitudes of the respondents.
6.	Questions aimed at determining the degree of readiness of the respondents to commit illegal acts, identifying attitudes towards offenders, as well as their own unlawful behavior.	“Is it always necessary to comply with the law?”, “Give an example when it is possible to violate a legal norm”, “Is it possible to violate a legal norm if there is no punishment for its violation?”, “Have you ever broken the law?”, “What crime do you consider the most serious and unacceptable? Do you consider it fair if such a crime is committed? What punishment do you believe is fair/appropriate if such a crime is committed?” “In what case would you most likely to commit such a crime?	There have been identified and generalized situations when the respondents expressed their willingness to commit an offense.The difference has also been revealed in relation to persons committing offenses and their own illegal behavior in similar situations.

* It is worth noting that the interview procedure and assessment of its results deserve a separate publication, therefore, only a general scheme of its implementation is presented in the framework of this article.

**Table 2:** An example of an algorithm of designing of the questionnaire items.

Interview Questions	Interview Example	Items (Statements) of the Questionnaire
The respondent is asked to name the human rights that are known to him or her. Then, each of the named categories of rights is discussed in detail separately with the interviewer. If the respondent finds it difficult to independently identify and disclose the categories of rights, the interviewer announces the official wording of the normative text to him, then suggests discussing the norm. An example (from the field of personal rights and freedoms). “Everyone has the right to liberty and security of person”, “Everyone who is legally on the territory of the Russian Federation has the right to move freely, choose a place of stay and residence. Everyone can freely travel outside the Russian Federation and return without hindrance. ”	*Interviewer*: What personal rights are you aware of? Do you consider personal rights significant for yourself? *Respondent R.S.*: Of course, personal rights are the most significant. The right to freedom of movement, freedom of conscience, thought, expression, the right to privacy. *Interviewer*: Are you ready to restrict your personal rights, for example, the right to freely travel abroad, if required by the civil service? *Respondent R.S.*: I would not want to be in a situation where my personal rights will have to be limited, but in certain conditions, if it is required for the common good, restrictions are possible. Perhaps I would agree to deprive myself of the possibility of going abroad, for the sake of service, in order to have additional social guarantees such as accommodation or early retirement.	I am well aware of my personal rights; I am well aware that everyone has the right to life and health, freedom of speech and thought, freedom of religion, freedom of conscience, right to privacy, the right to freedom of movement; I am ready to restrict part of my personal rights, if needed for the development of society. I am ready to limit my right to travel, to refuse the opportunity to freely travel outside my country and choose my place of residence if in return I receive sufficient material compensation or social guarantees. I am ready not to go abroad and live on the territory of only one country, if the state provides me with everything necessary and creates good conditions for my life.

**Table 3:** Total Variance Explained.

Factor	Eigenvalues	Percent of Variance	Cumulative Percent
1	14,560	25,999	25,999
2	5842	10,433	36,432
3	4894	8739	45,171
4	3707	6619	51,790
5	2848	5085	56,875
6	2736	4886	61,761

**Table 4:** Factor loadings (Varimax rotation, Principal component).

Item in the Initial Version of the Instrument	Item in the Final Version of the Instrument	Subscale	Factor					
			1	2	3	4	5	6
1	1	Awareness of Rights and Freedoms (1)	**0.794**	0.167	0.109	−0.042	−0.05	0.209
2	2		**0.707**	0.321	−0.002	0.106	0.065	−0.021
3	-	Personal Significance of Personal Rights and Freedoms (2)	item excluded					
4	3		−0.119	−0.093	0.054	−0.146	−0.078	**0.713**
5	-		item excluded					
6	4		0.013	0.07	0.19	−0.095	0.195	**0.726**
7	5		0.105	0.019	0.076	−0.149	0.092	**0.753**
8, 9	-		items excluded					
10	6		−0.023	0.049	−0.138	0.035	0.093	**0.673**
11	7		−0.012	−0.002	−0.036	0.218	0.128	**0.597**
12, 13, 14, 15, 16	-		items excluded					
17	8	Awareness of Rights and Freedoms (1)	**0.766**	−0.142	0.054	−0.178	0.15	−0.051
18	9		**0.815**	0.02	0.223	−0.084	0.093	−0.074
19	-	Personal Significance of Economic Rights and Freedoms (3)	item excluded					
20	10		0.136	−0.146	0.071	0.136	**0.805**	0.052
21	11		0.069	−0.003	0.051	0.079	**0.709**	0.181
22	-		item excluded					
23	12		−0.055	0.216	0.245	−0.034	**0.759**	−0.044
24	13		−0.006	0.104	0.155	−0.110	**0.732**	0.187
25	14		0.049	0.291	0.109	0.111	**0.777**	0.089
26	15	Awareness of Rights and Freedoms (1)	**0.761**	0.095	0.342	0.051	−0.08	−0.083
27	16		**0.672**	0.253	0.40	0.050	−0.008	−0.074
28	-	Personal Significance of Political Rights and Freedoms (4)	item excluded					
29	17		0.228	0.269	**0.817**	0.106	0.119	−0.124
30	18		0.167	0.227	**0.821**	0.077	0.221	−0.057
31	19		0.145	0.053	**0.857**	0.082	0.133	0.097
32	20		0.118	0.092	**0.771**	−0.078	0.191	0.155
33	21		0.24	0.326	**0.678**	−0.005	0.037	0.045
34, 35	-		items excluded					
36	22	Awareness of Rights and Freedoms (1)	**0.661**	0.510	0.086	0.015	0.082	0.039
37	23		**0.536**	0.580	0.219	0.17	0.179	−0.041
38, 39	-	Personal Significance of Social Rights and Freedoms (5)	items excluded					
40	24		0.228	**0.828**	0.139	0.088	0.217	0.020
41	25		0.175	**0.860**	0.250	−0.020	0.126	−0.038
42	26		0.265	**0.848**	0.213	0.112	0.080	0.044
43	27		0.129	**0.799**	0.194	0.176	−0.06	0.040
44	28	Awareness of Rights and Freedoms (1)	**0.792**	0.207	0.078	0.254	−0.065	0.017
45	29		**0.672**	0.299	0.004	0.370	0.110	−0.009
46	-	Personal Significance of Cultural Rights and Freedoms (6)	item excluded					
47	30		0.010	−0.085	−0.002	**0.801**	0.057	−0.069
48	31		−0.037	0.058	0.089	**0.714**	0.254	−0.159
49, 50, 51, 52	-		items excluded					
53	32		0.092	0.105	0.056	**0.875**	0.039	0.001
54	33		0.066	0.104	0.088	**0.849**	−0.108	0.047
55	34		0.076	0.190	−0.069	**0.795**	−0.032	0.021
56	-		item excluded					

Note: The highest factor loadings for each item are in bold.

## Supplementary Materials

The following are available online at https://www.mdpi.com/2076-328X/10/5/89/s1.
