# Supplementary material for: Survey for Assessment of a Person’s Legal Consciousness: Development and Preliminary Validation
Source: Behav Sci (Basel). 2020 May 12;10(5):89. doi: 10.3390/bs10050089 (PMC7288189; doi:10.3390/bs10050089)
Supplement: Supplementary file 1 [file behavsci-10-00089-s001.pdf]

Supplementary A

The list of the statements included into the initial questionnaire (56 items)

1. I am well aware of my personal rights.
2. I am well aware that every individual has the right to life and health, freedom of thought and speech, freedom of conscience, freedom of private life, freedom of movement.
3. It is important for me that all people are granted the same rights by birth, irrespective of the family and environment they are born in,
4. I am ready to waive particular personal rights, if it benefits the society's development.
5. It is important for me to have the right to choose the place to reside in, travel within the country, and leave the country.
6. I am ready to waive my freedom of movement, renounce my freedom to leave the country, and choose the place to reside in, if granted fair material remuneration and social welfare.
7. I am ready not to travel abroad and live within my motherland, if the state provides me with every sufficient need and decent living conditions.
8. It is important for me to have my private and family life secured, secrecy of my correspondence and line security maintained.
9. I am ready to give consent for access to particular aspects of my private life (such as correspondence, contacts, family life) for material remuneration and additional social welfare.
10. I am ready to give consent for access to particular aspects of my private life (i.e., correspondence, contacts, family life) to any special services, if it is necessary for the public wealth, maintenance of peace and security reasons.
11. Under no circumstances am I ready to restrict my right to private life.
12. It is important for me to have the liberty of thought and speech, freedom of conscience, to be free to express my opinion and to seek information without censorship.
13. I am ready to give up my freedom of thought and speech, if granted material remuneration and social welfare.
14. I am ready to give consent to Internet censorship if it benefits the social and cultural development.
15. Under no circumstances am I ready to give up my freedom of thought and speech, freedom of conscience, or restrict them in any way.
16. I am ready to live in the state with an established religion and join it on a mandatory basis, if the state grants me decent material remuneration and social welfare.
17. I am well aware of my economic rights.
18. I am well aware that every individual has the freedom to possess, use, and dispose the property based on the right to property; the right to labor and entrepreneurship.

19. It is important to me that I have the right to possess, use and dispose my property on the basis of the right to property, to buy property freely, to come into inheritance and to propagate what I possess on legal grounds.
20. I am ready to yield my right to property (e.g., houses, apartments , suburban houses, vehicles), if the state provides me with everything necessary and guarantees a decent standard of living (free education, free high-quality medicine, decent housing, well-paid job, decent retirement pension, etc.).
21. Under no circumstances am I ready to yield or restrict my right to property.
22. It is important to me that I have the right to labor (to define freely a form of activity, maintain business activity, or abstain from any labor).
23. I am ready to waive my freedom to labor, to be obliged to work and to bear responsibility for parasitism, if the state provides me with the better-paid job.
24. I am ready to waive the right not to work and to be obliged to labor, if granted the decent welfare - retirement pension, free medicine, and other social guarantees.
25. Under no circumstances am I ready to waive my right to labor and abstain from it.
26. I am well aware of my political rights.
27. I am well aware that everyone has the right to elect and be elected for authorities, the right to participate in a rally, to join a trade union, the freedom of peaceful assembly and association, to access the civil service.
28. It is important for me that I can elect representatives for bodies of authority and be elected as well, that I can affect governance directly and through elected bodies.
29. I am ready to waive the right to elect for material remuneration.
30. I am ready to waive the right to be elected for material remuneration and substantial social guarantees.
31. I am ready to waive the right to effect governance and display activism (i.e., to elect, to run for bodies of authorities), if I am fully satisfied with the level of living the state offers.
32. I am ready to abstain from participating in political (rallies, demonstrations) and social activities, if granted a decent standard of living.
33. Under no circumstances am I ready to waive my political rights and my citizenship.
34. It is important to me that no one can oblige me to join a trade union, an association or a political party.
35. I am ready to oblige to join a party, even though I do not share its political stance, if it benefits my wealth and career progress and guarantees financial reward.
36. I am well aware of my cultural rights.
37. I am well aware that an individual has the right to creative freedom, academic freedom, and access to culture.
38. It is important to me to have the right to creative and academic freedom, as well as to choose the art freely.
39. It is important for me to have the access to culture, to become acquainted with world and human history and culture, as well as the culture of my country, my area, and my community.

40. I am ready to waive the right to creative freedom, ready to write verses, songs, books, pictures within the framework of the state ideology, if it benefits my wealth and provides me substantial social guarantees.
41. Being a teacher or a lecturer, I would be ready to waive my academic freedom and build the learning process within the framework of the state ideology and school of the sciences, if it benefits my wealth and provides me substantial social guarantees.
42. I am ready to abstain from the access to culture, i.e., visiting museums, theaters. exhibitions, pieces of architecture, historical and cultural monuments, if granted high standard of living and substantial social guarantees.
43. Under no circumstances am I ready to waive my cultural rights.
44. I am well aware of my social rights.
45. I am well aware that an individual has the right to social security, namely free medical care and education, retirement pension, social guarantees and benefits for the disadvantaged, disabled, orphans, infants and others.
46. It is important for me to have the right to free education and medical care.
47. I am ready to waive the right to free medical care, if provided with a well-paid job.
48. I am ready to waive the free pre-school and school education, if provided with a well-paid job.
49. It is important to me to be liable for retirement pension.
50. I am ready to waive the right to the retirement pension, provided that I have benefits as a working individual to allow a decent retirement myself.
51. It is important to me that the state supports maternity and provides maternity and parental leave.
52. It is important to me to have the right to free emergency medical treatment.
53. Under no circumstances am I ready to waive free medical care.
54. Under no circumstances am I ready to waive the retirement pension.
55. Under no circumstances am I ready to waive free education.
56. Under no circumstances am I ready to waive social security and benefits, due to the delivery of a child, illness or disabilities.

Supplementary B

Legal Consciousness Questionnaire.

Guidelines*:* Dear participant,

Read the following statements carefully and express the level of agreement/disagreement, where: 1 – completely disagree; 2 – disagree; 3 – partly disagree; 4 – neutral; 5 – partly agree; 6 – agree; 7 – completely agree.

№	Statement	Degree of Agreement
1	I am well aware of my personal rights.	
2	I am well aware that every individual has the right to life and health, freedom of thought and speech, freedom of conscience, freedom of private life, freedom of movement.	
3	I am ready to waive particular personal rights, if it benefits the society's development.	

4	I am ready to waive my freedom of movement, to renounce my freedom to leave the country, and choose the place to reside in, if granted fair material remuneration and social welfare.
5	I am ready not to travel abroad and to live within my motherland, if the state provides me with every sufficient need and decent living conditions.
6	I am ready to give consent for access to particular aspects of my private life (i.e., correspondence, contacts, family life) to any special services, if it is necessary for the public wealth, maintenance of peace and security reasons
7	Under no circumstances am I ready to restrict my right to private life.
8	I am well aware of my economic rights.
9	I am well aware that every individual has the freedom to possess, use, and dispose the property based on the right to property; the right to labor and entrepreneurship.
10	I am ready to yield my right to property (e.g., houses, apartments, suburban houses, vehicles), if the state provides me with everything necessary and a decent standard of living (free education, free high-quality medical care, decent housing, well-paid job, decent retirement pension, etc.).
11	Under no circumstances am I ready to yield or restrict my right to property.
12	I am ready to waive my freedom to labor, to be obliged to work and bear responsibility for parasitism, if the state provides me with the better-paid job.
13	I am ready to waive the right not to work and to be obliged to labor, if granted the decent welfare - retirement pension, free medicine, and other social guarantees.
14	Under no circumstances am I ready to waive my right to labor and abstain from it.
15	I am well aware of my political rights.
16	I am well aware that everyone has the right to elect and be elected for authorities, the right to participate in a rally, join a trade union, the freedom of peaceful assembly and association, to access the civil service.
17	I am ready to waive the right to elect for material remuneration.
18	I am ready to waive the right to be elected for material remuneration and substantial social guarantees.
19	I am ready to waive the right to affect governance and display activism (i.e., to elect, to run for bodies of authorities), if I am fully satisfied with the level of living the state offers.
20	I am ready to abstain from participating in political (rallies, demonstrations) and social activities, if granted a decent standard of living.
21	Under no circumstances am I ready to waive my political rights and my citizenship.
22	I am well aware of my cultural rights.
23	I am well aware that an individual has the right to creative freedom, academic freedom, and access to culture.
24	I am ready to waive the right to creative freedom, ready to write verses, songs, books, pictures within the framework of the state ideology, if it benefits my wealth and provides me substantial social guarantees.
25	I am ready to waive the right to creative freedom, ready to write verses, songs, books, pictures within the framework of the state ideology, if it benefits my wealth and provides me substantial social guarantees.
26	I am ready to abstain from the access to culture, i.e., visiting museums, theaters. exhibitions, pieces of architecture, historical and cultural monuments, if granted high standard of living and substantial social guarantees.
27	Under no circumstances am I ready to waive my cultural rights.
28	I am well aware of my social rights.
29	I am well aware that an individual has the right to social security, namely free medical care and education, retirement pension, social guarantees and benefits for the disadvantaged, disabled, orphans, infants and others.
30	I am ready to waive the right to free medical care, if provided with a well-paid job.
31	I am ready to waive the free pre-school and school education, if provided with a well-paid job.
32	Under no circumstances am I ready to waive free medical care.
33	Under no circumstances am I ready to waive the retirement pension.

34	Under no circumstances am I ready to waive free education.

The structure of the questionnaire

Items	Category of the Rights	Legal Awareness	Significance of Rights
1–7	Personal	1, 2	3, 4, 5, 6, 7
8–14	Economic	8, 9	10, 11, 12, 13, 14
15–21	Political	15, 16	17, 18, 19, 20, 21
22–27	Cultural	22, 23	24, 25, 26, 27
28–34	Social	28, 29	30, 31, 32, 33, 34

The key to the questionnaire.

Subscale 1. Overall awareness of the constitutional rights (10 items) – 1, 2, 8, 9, 15, 16, 22, 23, 28, 29.

Personal rights awareness – 1, 2
Economic rights awareness – 8, 9
Political rights awareness – 15, 16
Cultural rights awareness – 22, 23
Social rights awareness – 28, 29

Subscale 2. Personal Significance of the Personal Rights (5 items) – 3R, 4R, 5R, 6R, 7
Subscale 3. Personal Significance of the Economic Rights (5 items) – 10R, 11, 12R, 13R, 14
Subscale 4. Personal Significance of the Political Rights (5 items) – 17R, 18R, 19R, 20R, 21
Subscale 5. Personal Significance of the Social Rights (4 items) – 24R, 25R, 26R, 27
Subscale 6. Personal Significance of the Cultural Rights (5 items) – 30R, 31R, 32, 33, 34
